# Complete Genome Sequence of *Faecalibacillus intestinalis* JCM 34082, Isolated from Feces from a Healthy Japanese Female

**DOI:** 10.1128/MRA.01160-20

**Published:** 2020-12-10

**Authors:** Mitsuo Sakamoto, Nao Ikeyama, Atsushi Toyoda, Takumi Murakami, Hiroshi Mori, Moriya Ohkuma

**Affiliations:** a Microbe Division/Japan Collection of Microorganisms, RIKEN BioResource Research Center, Tsukuba, Ibaraki, Japan; b PRIME, Japan Agency for Medical Research and Development, Tsukuba, Ibaraki, Japan; c Advanced Genomics Center, National Institute of Genetics, Mishima, Shizuoka, Japan; University of Maryland School of Medicine

## Abstract

Here, we report the complete genome sequence of *Faecalibacillus intestinalis* JCM 34082, a member of the family *Erysipelotrichaceae* that was isolated from feces from a healthy Japanese woman. The genome assembly comprised 2,869,982 bp, with a G+C content of 29.8%.

## ANNOUNCEMENT

**FIG 1:**
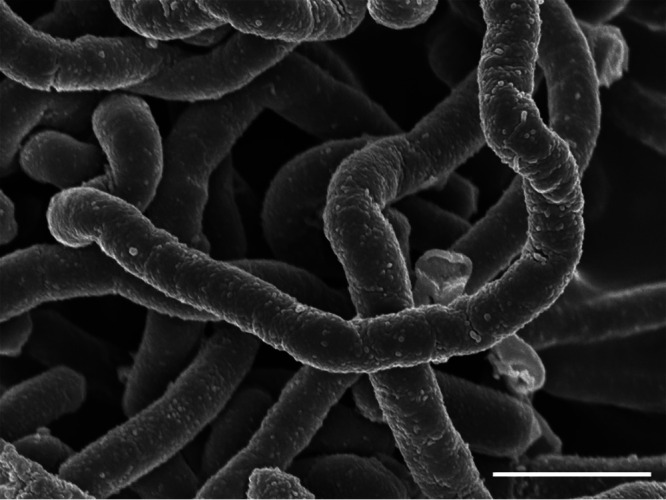
Electron micrograph of cells of *F. intestinalis* JCM 34082. The image was obtained by scanning electron microscopy. Bar, 1 μm. The morphology of cells after 4 days of culture on EG agar was observed using scanning electron microscopy (JEM-6340F; JEOL). Sample preparation for scanning electron microscopy has been described previously (7).

*Faecalibacillus* is a Gram-positive, obligately anaerobic long-rod-shaped genus of the family *Erysipelotrichaceae*. *Faecalibacillus intestinalis* was first isolated from human feces from healthy South Korean subjects (1). Many 16S rRNA gene sequences that appear to belong to this characteristic long bacillus, *F. intestinalis*, are found in public databases. Our isolate was identified as *F. intestinalis* based on the16S rRNA gene sequences, as well as the observed characteristic long bacillus form (Fig. 1). Here, we report the complete genome sequence of *F. intestinalis* JCM 34082.

This study was approved by the RIKEN Ethics Committee (approval number Tsukuba 27-1). A fecal sample was obtained from a healthy Japanese woman in her 40s in March 2019. An informed consent agreement was obtained from the volunteer before the experiment. A portion (0.5 g) of the fecal sample was suspended in 4.5 ml of prereduced phosphate-buffered saline. The diluted fecal sample was plated onto Eggerth-Gagnon (EG) agar (Merck) supplemented with 5% (vol/vol) horse blood (JCM medium 14) for 2 to 4 days of incubation at 37°C under an H_2_/CO_2_/N_2_ (1:1:8, by volume) gas mixture. The grown colonies were screened by partial 16S rRNA gene sequencing to identify and isolate *F. intestinalis* strain 14EGH31 (JCM 34082). The 16S rRNA gene of strain 14EGH31 was amplified by PCR with universal primers 27F and 1492R (2). After the sequencing, we performed a similarity search using NCBI nucleotide BLAST, and we identified strain 14EGH31 as *F. intestinalis* (100% similarity). *F. intestinalis* JCM 34082 was then grown in 500 ml of Gifu anaerobic medium (Nissui) for 4 days at 37°C to prepare the genomic DNA. The genomic DNA of *F. intestinalis* JCM 34082 was extracted by using a Genomic-tip 100/G kit (Qiagen) and lysing bacterial cells with Labiase (5.0 mg/ml; Cosmo Bio). The whole genome of *F. intestinalis* JCM 34082 was sequenced as follows. A SMRTbell library was constructed using a SMRTbell Express template preparation kit v2.0 (Pacific Biosciences) according to the manufacturer's protocol. The sequencing library was size selected using the BluePippin system (Saga Science) with a minimum fragment length cutoff of 20 kbp. One single-molecule real-time (SMRT) cell (1M v3 LR tray) was run on the PacBio Sequel System with binding kit v3.0, sequencing kit v3.0, and 1,200-min movies, yielding a total of 882,011 subreads (total, 11,716,679,888 bp; *N*_50_, 23,089 bp). Low-quality subreads and adapters were filtered using SMRT Link v8.0.0.84512 (Pacific Biosciences), and then qualified reads were assembled using the Canu v2.0 assembler program (3) with default settings. In addition, genomic DNA was fragmented to an average size of 600 bp with the M220 DNA-shearing system (Covaris, Inc.). A paired-end library was constructed with a TruSeq DNA PCR-free library preparation kit (Illumina) and was size selected on an agarose gel using a Zymoclean large-fragment DNA recovery kit (Zymo Research). The final library was sequenced on the Illumina HiSeq 2500 sequencer with a read length of 250 bp. Read sequences comprising 5,585,708 raw reads were used to improve the assembled sequence with the Pilon v1.23 software tool (4). This assembly resulted in a complete genome sequence of 2,869,982 bp, with a G+C content of 29.8% and 4,018-fold coverage, containing 2,948 protein-coding sequences, 66 tRNAs, and 27 rRNAs detected by using the DFAST v1.2.4 pipeline with the Prodigal option (https://dfast.nig.ac.jp) (5, 6). Default parameters were used for all software except where otherwise noted.

### Data availability.

The complete genome sequence of *F. intestinalis* JCM 34082 has been deposited in DDBJ/ENA/GenBank under the accession number AP024085. Raw sequence reads have been deposited in the Sequence Read Archive (SRA) under BioProject number PRJDB10321 and the accession numbers DRR248858 (PacBio) and DRR248859 (Illumina).
